# Sensor-Based Tracking and Localization of In-Line Inspection Tools in Oil and Gas Pipelines: A Review

**DOI:** 10.3390/s26165030

**Published:** 2026-08-07

**Authors:** Jianfeng Zheng, Bingfeng Ju, Anyu Sun

**Affiliations:** 1 School of Mechanical Engineering, Zhejiang University, Hangzhou 310030, China; zhengjf@pipechina.com.cn; 2 PipeChina Research Institute, Tianjin 300450, China

**Keywords:** pipeline inspection gauge, in-line inspection, ELF magnetic sensing, distributed acoustic sensing, sensor fusion, tracking and localization, intelligent sensing

## Abstract

Accurate tracking and localization of in-line inspection (ILI) tools are essential for mileage calibration, defect mapping, and blockage prevention in oil and gas pipelines. This review summarizes sensor-based approaches for external ILI-tool localization, emphasizing how sensing physics, deployment geometry, and signal interpretation determine practical performance. Extremely low-frequency (ELF) magnetic tracking is first examined through dipole modeling, sensor evolution, and weak-signal recovery under steel-pipe and soil shielding. Distributed fiber-optic sensing is then reviewed as a continuous-tracking alternative, with attention to fading mitigation, spatiotemporal denoising, and trajectory extraction from distributed acoustic sensing data. Acoustic arrays and hybrid schemes are discussed as complementary options for subsea or cable-free environments. Finally, the review assesses how data fusion and lightweight artificial intelligence (AI) can improve robustness while noting unresolved issues in field data availability, edge computing, and uncertainty quantification. The synthesis indicates that next-generation ILI tracking should combine heterogeneous sensing, physics-aware signal processing, and deployment-aware model design rather than rely on a single high-sensitivity sensor.

## 1. Introduction

With the sustained growth in global energy demand, oil and gas pipelines are rapidly extending toward larger diameters, higher pressures, longer distances, and more complex geographical environments. To detect corrosion, cracks, and mechanical damage in pipe walls, both ILI and external nondestructive testing have advanced rapidly through developments in sensing technologies and AI-assisted analysis methods [1,2,3,4,5]. For long-distance buried pipelines, ILI is widely used, making accurate tracking and localization of the inspection tool essential for defect mapping and integrity decision-making.

An ILI tool, or pipeline inspection gauge (PIG), travels inside underground pipelines at uncertain speeds, and tracking and localization refer to the technologies used to obtain its real-time position from the ground surface. Accurate tracking and localization are not only essential for the rapid response to abnormal tool blockage, but also directly affect the reliability of mileage calibration, defect spatial mapping, and integrity assessment results derived from ILI data [1,2].

**Figure 1:**
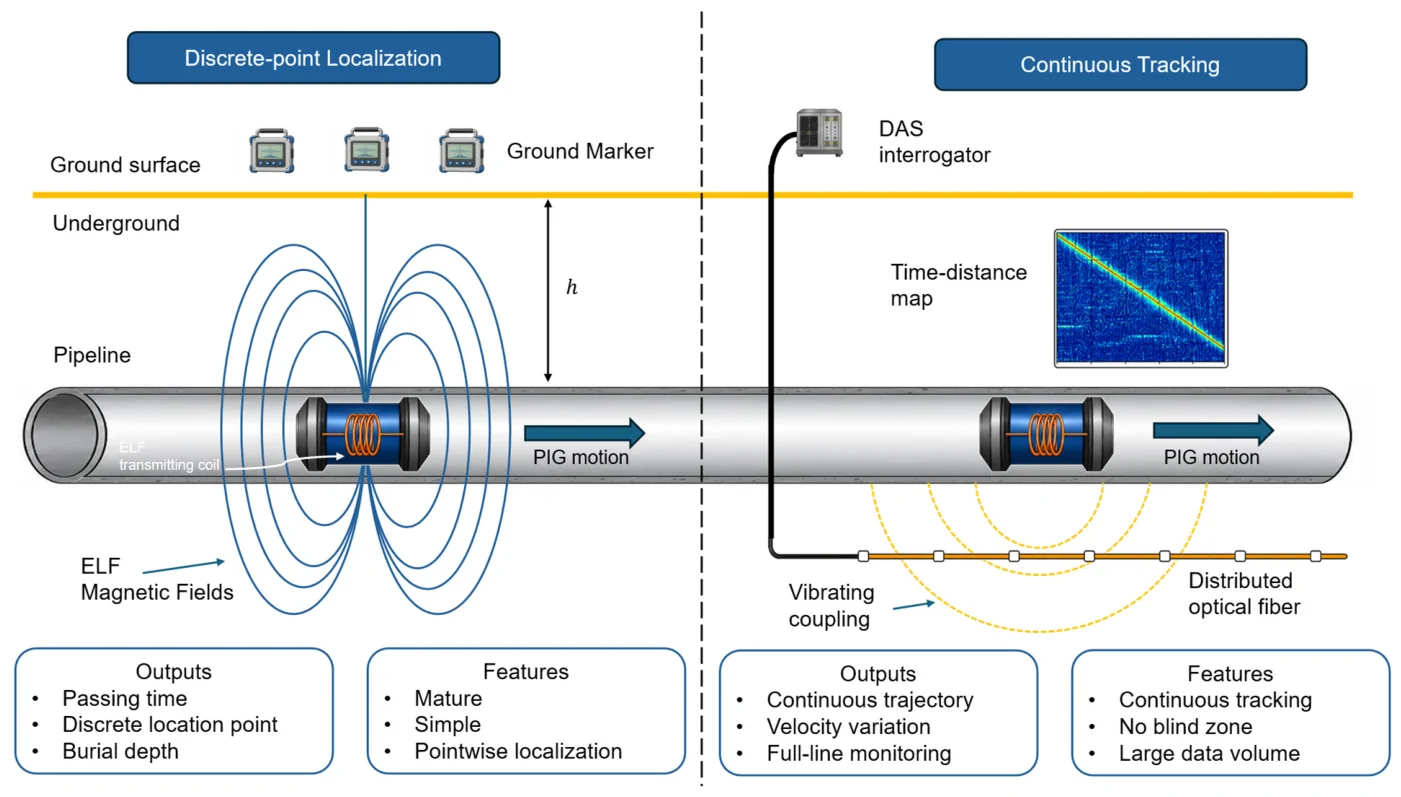
Sensor modalities and tracking/localization outputs for pipeline ILI tools.

From the perspective of sensing architecture, existing tracking and localization methods for pipeline ILI tools can be broadly divided into discrete node localization and full-line continuous tracking. Discrete localization mainly relies on ground markers deployed along the pipeline to capture electromagnetic or acoustic signatures generated when the ILI tool passes specific measurement points, thereby obtaining information such as passage time, discrete position, and burial depth. Continuous tracking, in contrast, uses distributed optical fibers laid alongside the pipeline to continuously sense vibration responses induced by tool motion over long distances and to further invert velocity variations and operating trajectories [6,7]. The basic sensing principles, system configurations, and major outputs of the two approaches are shown in Figure 1.

However, the two categories face different bottlenecks. Discrete localization is highly susceptible to signal penetration attenuation caused by steel pipes and overburden soil [8,9], whereas continuous tracking is more vulnerable to environmental vibration noise, interference fading, and false alarms [6,10,11]. Consequently, performance gains achievable through improvements in conventional single sensors and linear signal processing alone are approaching their limits, making further enhancement increasingly difficult [9,10,12].

Previous reviews have mainly addressed pipeline safety monitoring and integrity assessment at a broad application level [1,2], machine-learning methods and event detection for distributed acoustic sensing-based pipeline surveillance [10,13], magnetic-sensor development [14,15], or intelligent magnetic-flux-leakage inspection and defect evaluation [16]. These studies provide valuable modality- or task-specific summaries, but they do not jointly examine external ILI-tool tracking and localization across discrete and continuous sensing architectures. In contrast, this review adopts a sensor-centric and cross-modal perspective that integrates ELF magnetic sensing, distributed fiber-optic sensing, acoustic and hydrophone localization, weak-signal processing, sensor fusion, and emerging AI methods within a common sensor-system framework. Particular attention is given to how sensing physics, observable quantities, coverage continuity, deployment geometry, signal-processing pipelines, deployment burden, evidence relevance, and validation maturity jointly determine practical localization performance. This integrated cross-modal comparison constitutes the principal novelty of the review and provides a system-oriented roadmap for the development of next-generation ILI-tool tracking and localization systems.

The main contributions of this review are fourfold. First, it reframes external ILI/PIG tracking as an integrated sensor-system problem rather than as an auxiliary operation. Second, it compares discrete magnetic localization, distributed fiber-optic tracking, acoustic and hydrophone localization, and hybrid fusion using common system-level criteria. Third, it connects front-end sensor evolution with weak-signal processing and intelligent interpretation, showing how hardware sensitivity, signal reconstruction, and AI-assisted localization jointly affect field robustness. Fourth, it distinguishes mature engineering solutions from emerging frameworks such as physics-informed learning, topology-aware fusion, and digital twins, thereby identifying practical research gaps for high-precision and deployable tracking systems.

To improve the transparency of the review process, the literature scope and selection approach are briefly described here. Relevant studies were identified through targeted searches of the Web of Science Core Collection, Scopus, IEEE Xplore, Engineering Village–Compendex, and ScienceDirect databases. The review focused mainly on peer-reviewed journal articles and conference papers published between 2005 and 2026, while earlier seminal studies were retained where necessary to establish the theoretical foundations of electromagnetic modeling, distributed fiber-optic sensing, time-delay estimation, state estimation, and weak-signal processing.

The targeted searches used broad combinations of terms related to PIGs or ILI tools, tracking and localization, ELF magnetic sensing, distributed fiber-optic sensing, acoustic or hydrophone localization, sensor fusion, and emerging AI methods. Priority was given to studies directly addressing external PIG tracking and localization or reporting relevant sensing principles, sensor configurations, signal-processing methods, system architectures, quantitative performance, and experimental or field-validation evidence. Studies from related pipeline-sensing applications were included only when their sensing mechanisms, physical models, or data-processing methods were considered transferable to external ILI-tool localization. Duplicate records, abstract-only publications, nontechnical reports, studies focusing exclusively on internal defect detection, and general pipeline-monitoring studies without transferable relevance to external ILI-tool localization were excluded. Emerging methods with limited direct validation were treated as prospective approaches.

The selected studies were subsequently organized according to publication year, primary technical category, coverage continuity, observable features, deployment burden, evidence relevance, and validation maturity. Validation maturity was classified as simulation or theoretical analysis, laboratory experiment, pipeline-loop or full-scale controlled test, short field test, and operational-pipeline deployment. For the descriptive statistical analysis, review articles, textbooks, and general methodological references used only to establish theoretical foundations were excluded. Each application-oriented study was assigned to one primary technical category according to its principal sensing input or system architecture. When a study reported more than one validation stage, only the highest validation level was counted to avoid duplicate classification. Table 1 summarizes the principal cross-modal tradeoffs among the reviewed sensing strategies and includes representative quantitative performance and validation information where available. Because the cited studies use different pipeline geometries, sensor configurations, performance definitions, and test conditions, the reported values are not directly comparable.

To provide a brief quantitative overview of the literature included in this review, 30 application-oriented original studies were included in the descriptive analysis. Of these studies, 25 (83.3%) were published between 2021 and 2026, indicating a marked increase in research activity in recent years. Magnetic or electromagnetic approaches accounted for four studies (13.3%), distributed fiber-optic approaches for eight studies (26.7%), acoustic or acoustic-emission approaches for four studies (13.3%), pressure-, hydraulic-, or network-state-based approaches for six studies (20.0%), and multimodal, data-driven, or system-level approaches for eight studies (26.7%). The reviewed literature included simulation or data-based studies, laboratory and controlled experiments, and field or operational-pipeline investigations; however, validation under long-distance operational conditions remained comparatively limited.

## 2. Sensor Foundations of ELF Electromagnetic Tracking

**Figure 2:**
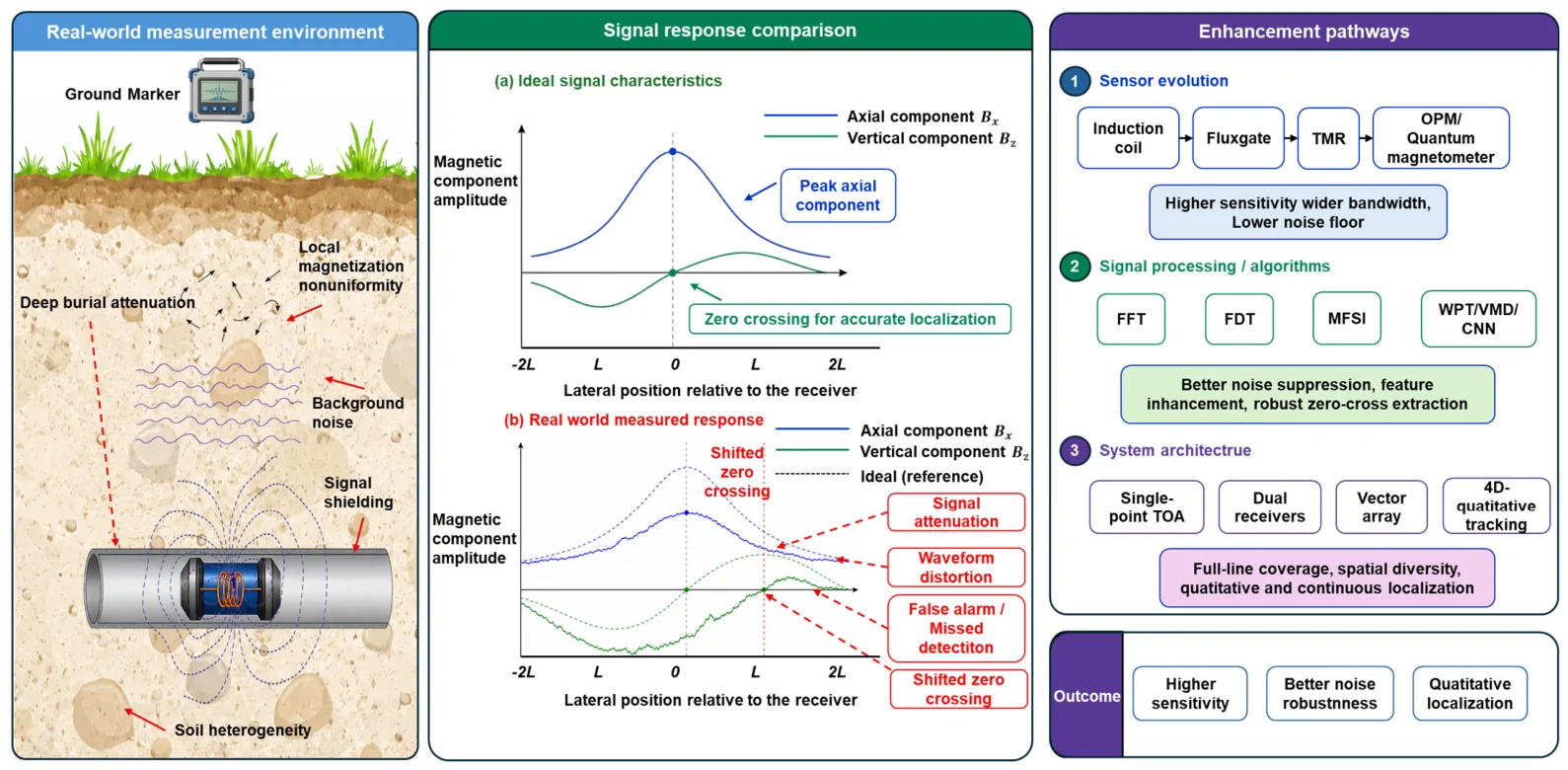
ELF electromagnetic sensing for pipeline ILI tracking.

ELF electromagnetic tracking is fundamentally a magnetic sensing problem in which a transmitter mounted on the ILI tool interacts with magnetic-field receivers deployed on the ground surface. As the emitted signal propagates through the steel pipe wall, soil cover, and complex geological media, it is jointly affected by eddy-current shielding, medium inhomogeneity, and environmental electromagnetic noise. Consequently, the received signal deviates from the ideal magnetic-dipole response through amplitude attenuation, waveform distortion, zero-crossing shifts, and degraded signal-to-noise ratio. For this reason, the performance of ELF tracking depends not only on localization algorithms, but also on the entire sensing chain, including physical modeling, sensor sensitivity, receiver configuration, and weak-signal processing. The practical sensing environment, the discrepancy between ideal and measured signals, and the corresponding enhancement paths are shown in Figure 2 [18,19].

### 2.1. Physical Sensing Model of ELF Tracking Systems

#### 2.1.1. Classical Time-Harmonic Magnetic Dipole Representation

In pipeline ILI-tool tracking and localization systems based on ELF electromagnetic waves, the transmitting coil installed on the tool is driven by an extremely low-frequency alternating current. Because the geometric dimensions of the transmitting coil are much smaller than both the wavelength of the ELF electromagnetic wave and the observation distance to the ground receiver, the source can be idealized in free space and nonmagnetic media as a time-harmonic magnetic dipole model [18,19].

A Cartesian coordinate system is established with the magnetic dipole center as the origin. The ILI tool is assumed to move along the pipeline axial direction (X-axis), with the magnetic moment m oriented parallel to the X-axis. Under the quasi-static electromagnetic-field approximation that neglects eddy-current effects in the pipe wall and soil, the magnetic-flux-density components at an arbitrary point P(x, y, z) can be derived from the Biot–Savart law as follows:

$$B_{x}=\frac{\mu_{0}m}{4\pi R^{5}}\left(2x^{2}-y^{2}-z^{2}\right)$$

$$B_{y}=\frac{\mu_{0}m}{4\pi R^{5}}\left(3xy\right)$$

$$B_{z}=\frac{\mu_{0}m}{4\pi R^{5}}\left(3xz\right)$$

where $\mu_{0}\;$denotes the vacuum permeability, m is the magnitude of the magnetic dipole moment, and $R=\sqrt{\left(x^{2}+y^{2}+z^{2}\right)}$^2^ is the straight-line distance from the observation point to the magnetic source.

Existing studies generally design the detection algorithms of ground reference locators on the basis of the above classical equations. Two key tracking characteristics can be inferred from the equations: when the receiver is located directly above the ILI tool, the axial component reaches its maximum while the vertical component crosses zero exactly; moreover, the curve exhibits a very steep slope near the zero-crossing point, which provides the key basis for precise localization of the tool [9,18,19].

#### 2.1.2. Limits of the Idealized Sensing Model

The idealized model above cannot fully capture practical engineering conditions. According to Maxwell’s equations, eddy currents in the conductive pipe wall generate an opposing magnetic field, thereby distorting the field produced by the transmitting coil. The classical formula for signal skin depth is given by [18,19]:

$$\delta =\sqrt{\frac{2}{\omega \mu \sigma }}$$

where ω is the angular frequency, μ is the magnetic permeability, and σ is the electrical conductivity. For high-strength pipeline steels such as X80, the large relative magnetic permeability μr sharply reduces the skin depth of ELF signals and causes signal attenuation to increase exponentially.

In addition, local magnetic nonuniformity in the pipe wall and variations in the electromagnetic parameters of deep soil can cause spatial shifts in the observed zero-crossing point and introduce nonstationary electromagnetic interference and Gaussian-like sensor noise into the signal. This makes the conventional time-domain voltage comparison method based on fixed thresholds highly prone to missed detections and false alarms in deeply buried sections [19,20,21].

### 2.2. Impact of Magnetic Sensor Evolution on Tracking Performance

Every improvement in electromagnetic tracking technology fundamentally depends on progress in front-end magnetically sensitive devices. After the ELF electromagnetic signal emitted by the ILI tool penetrates thick-walled steel pipes and deep soil cover, the ground-received signal is already severely attenuated, which requires the sensor to offer both excellent low-frequency response and extremely low thermal noise.

Traditional ground receivers rely on electromagnetic induction coils. As passive devices, their advantages include simple structure, high stability, and no need for external excitation. However, according to Faraday’s law of electromagnetic induction, the induced electromotive force is limited by the rate of magnetic-flux variation. For ELF signals around 20 Hz, the intrinsic rate of flux variation in the coil is very small, which imposes an inherent limitation on the detection of weak signals. Overcoming the thermal-noise limit by increasing signal strength usually requires a substantial increase in the number of coil turns and effective cross-sectional areas, thereby reducing the engineering portability of the equipment [14,15].

To overcome the sensitivity and size limitations of induction coils in ELF detection, active magnetometers such as fluxgate and tunneling magnetoresistance (TMR) sensors have attracted increasing attention. Fluxgate sensors directly measure low-frequency and quasi-static magnetic fields, but their performance is more appropriately characterized using frequency-dependent noise spectral density rather than a universal detection limit. However, their performance depends on the core design, excitation mode, measurement bandwidth, and test environment, while probe size and excitation power remain important engineering constraints [14]. With the development of micro- and nanofabrication technologies, TMR sensors, which operate through spin-dependent quantum tunneling, have attracted increasing interest because of their chip-scale dimensions and potentially low power consumption. For ELF weak signals, the performance of TMR sensors is strongly dependent on measurement frequency, device configuration, bias conditions, readout electronics, and low-frequency 1/f noise. Optimized TMR devices have reported detectivity of approximately 5 $pT/\sqrt{Hz}$ at 1 Hz under specified laboratory conditions [15]. In a representative laboratory study, 5 kHz AC modulation, impedance compensation, and gradiometric measurement improved the signal-to-noise ratio by 25.3 dB at 1 Hz in an unshielded environment [22]. These characteristics indicate the potential of TMR sensors for compact magnetic-vector arrays and spatial-gradient measurements in portable ground receivers. However, these reported values describe sensor-level performance under specific laboratory conditions and should not be interpreted as equivalent detection depth or localization accuracy in buried or subsea pipelines. Pipeline-level performance also depends on pipe-wall attenuation, transmitter strength, standoff distance, environmental magnetic interference, sensor orientation, array geometry, and signal-processing methods.

Although fluxgate and TMR sensors have improved weak-field measurement capabilities, ELF signals may remain difficult to detect in deeply buried or subsea pipelines because of pipe-wall attenuation, standoff distance, environmental magnetic interference, and sensor noise. Optically pumped atomic magnetometers (OPMs) provide a potential alternative, and selected systems have reported femtotesla-level noise spectral density under carefully controlled operating conditions [23,24]. However, such values are strongly dependent on measurement bandwidth, atomic-vapor-cell temperature, optical-pumping parameters, magnetic shielding or active field compensation, and the operating magnetic-field range. In particular, the highest sensitivities associated with spin-exchange-relaxation-free operation generally require carefully controlled near-zero-field laboratory conditions. Therefore, femtotesla-level laboratory sensitivity should not be interpreted as equivalent field performance for external PIG tracking. The applicability of OPMs to buried and subsea pipelines remains prospective and requires pipeline-specific experimental validation.

Table 2 summarizes the evolution and engineering characteristics of ELF magnetic sensors for external ILI-tool tracking.

Overall, the evolution of ELF magnetic sensors has progressed from mature passive induction coils to active fluxgate and TMR devices and, more recently, prospective quantum sensors such as OPMs. This evolution reflects not only improvements in sensitivity and miniaturization, but also changing engineering tradeoffs in bandwidth, frequency-dependent noise, power consumption, device size, environmental robustness, and requirements for excitation, shielding, or field compensation. These device-level characteristics, together with pipe-wall attenuation, transmitter strength, standoff distance, and receiver-array geometry, define the practical application boundaries of each sensor. Therefore, laboratory specifications obtained at particular frequencies and under controlled conditions should not be interpreted directly as universal detection depths or localization accuracies. Future development should emphasize application-specific validation and balanced optimization rather than nominal sensitivity alone.

### 2.3. Weak-Signal Extraction and Algorithmic Evolution Under Extreme Operating Conditions

As the operating pressure of high-grade, large-diameter gas pipelines increases, the running speed of the ILI tool can exceed 10 m/s. Under these conditions, the time window during which the ELF transmitter passes through the sensing region of the ground receiver is significantly compressed, and the originally continuous sinusoidal signal degenerates into a millisecond-level transient pulse [8]. Under the combined effects of pipe-wall eddy-current interference and delay drift, the signal exhibits the coexistence of strong and weak components, short duration, and enhanced nonstationarity, making conventional threshold-based judgment and frequency-domain analysis inadequate for recognizing extremely weak signals [20,21,25,26]. Table 3 summarizes representative ELF tracking signal-processing pipelines and compares their engineering tradeoffs.

In practical field-detection environments, the raw signals captured by ground receiving arrays are often masked by high-amplitude power-frequency harmonics and nonstationary environmental noise. A staged denoising strategy is therefore required. First, adaptive notch filters are used to remove narrowband interference. Then, to eliminate spatial shifts caused by local magnetic nonuniformity of the pipe wall in the dual-receiver model, hierarchical feature reconstruction can be carried out by combining wavelet packet transform (WPT), variational mode decomposition (VMD), and one-dimensional deep networks. On the one hand, these methods improve the temporal resolution of zero-crossing detection for ELF pulses; on the other hand, they enhance system robustness to abrupt speed variations and background noise, enabling ELF tracking to evolve gradually from event triggering toward quantitative measurement [21,25,26,27,28].

### 2.4. Sensor Deployment and System-Level Localization Optimization

In early engineering applications, ground receivers were usually used only as simple trigger switches, and their principal limitation was that they could provide only one-dimensional time-of-arrival (TOA) information. This one-dimensional detection mode cannot infer the specific burial depth of the ILI tool, nor can it identify the lateral physical offset of the ground receiver. In densely populated underground urban pipeline networks or mountainous areas with rugged terrain, patrol personnel often find it difficult to position themselves exactly above the geometric centerline of the pipeline. Such unavoidable positional offsets can introduce significant localization errors and severely degrade positioning accuracy [8,9].

To overcome the limitations of one-dimensional physical detection, researchers first conducted extensive exploration in system hardware optimization and low-level signal processing. To address the problem that, in large-diameter, high-pressure pipelines, high-speed tool motion causes the ground ELF signal to appear as an extremely short-duration and extremely weak transient pulse, Piao et al. proposed a real-time tracking system based on orthogonal search-coil sensors and a fast-decision-tree (FDT) algorithm from the perspective of signal fusion. In a full-scale controlled test, the system used the envelope attenuation characteristics of the ELF signal to detect high-speed PIG passage under low-SNR conditions [8]. In addition, Song et al. improved acoustic vector sensing devices used in ground markers, providing another feasible sensing solution for discrete node detection [29]. Overall, subsequent studies on ground-marker systems and transmitter-side structural optimization have been reflected more in engineering-level enhancement than in a change in the localization paradigm itself.

To alleviate information loss caused by spatial dimensionality reduction, Long et al. proposed a four-dimensional tracking system and reported centimeter-level localization performance in a full-scale controlled test [9]. Instead of the isolated single-point observation mode, the system deploys two or more spatially separated receiving-coil arrays on the ground surface. By using a differential structure to capture the spatial variation and gradient information of weak magnetic-field vectors, the system jointly solves the four-dimensional state vector of the PIG using nonlinear least squares, namely the instantaneous axial position, lateral offset, absolute pipeline burial depth, and precise timestamp, thereby enabling quantitative characterization of tool operating states. As the core of this system, magnetic field sign integration (MFSI) theory introduces sign-function-weighted integration to effectively suppress noise and magnetic-field distortion, driving ELF tracking from qualitative judgment toward quantitative reconstruction.

## 3. Sensors for Continuous Tracking

Traditional electromagnetic tracking based on ground reference locators is essentially a discrete sensing mode in which the system can obtain the tool position only when the ILI tool passes beneath a specific marker, leaving long blind zones between markers [8,9,29]. Pipeline monitoring is therefore moving toward continuous sensing over the full line. Distributed fiber-optic sensing uses communication optical cables laid in the same trench as the pipeline and relies on phase-sensitive optical time-domain reflectometry to capture in real time the vibration signals generated by tool motion. This approach can provide continuous time–distance trajectories between discrete marker locations and has demonstrated long-range PIG tracking in a short field test, although its performance remains dependent on fiber deployment, vibration coupling, environmental noise, and interference fading [6,7].

**Figure 3:**
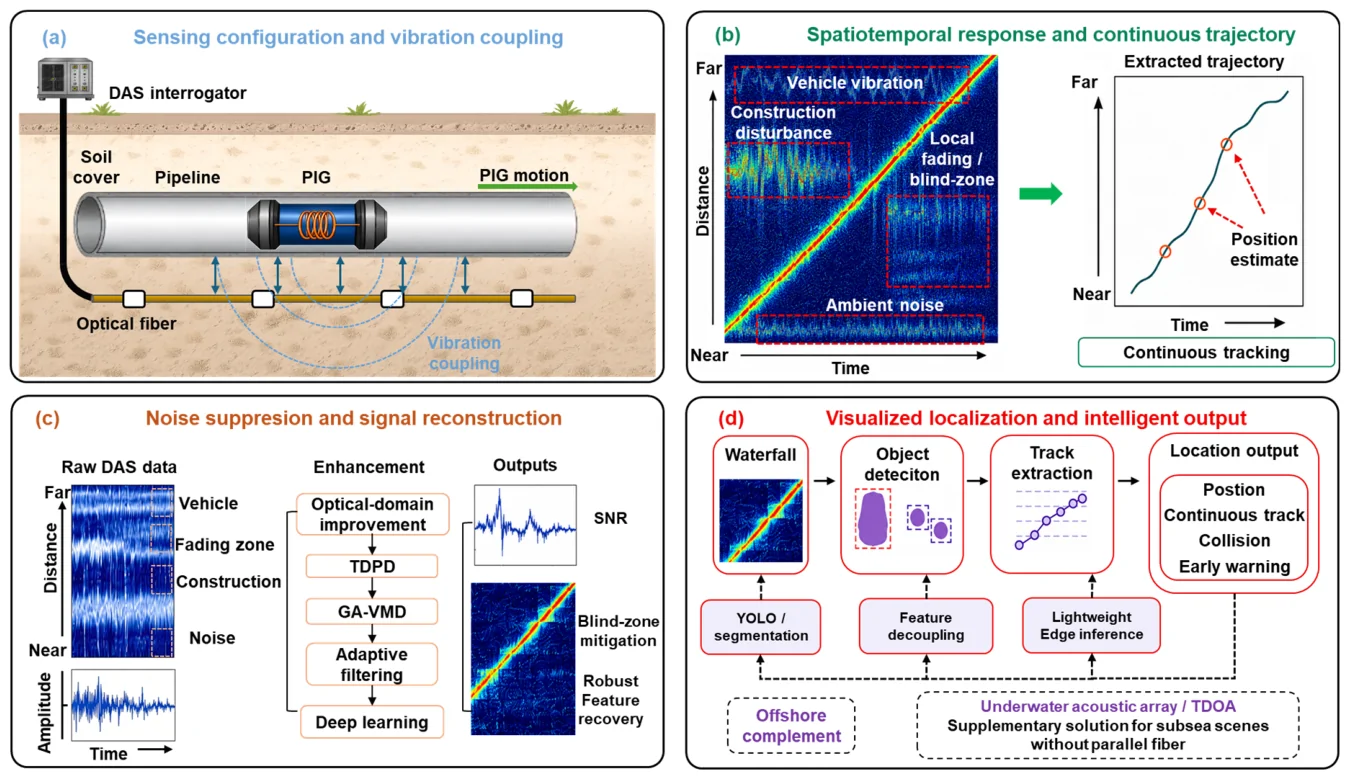
Distributed fiber-optic sensing for ILI tracking.

Distributed fiber-optic tracking uses optical cables along the pipeline as a continuous sensing medium. By acquiring vibration signals generated by the interaction of the ILI tool with the pipe wall, the medium, and pipeline auxiliary structures during operation, it forms a two-dimensional spatiotemporal response containing time and distance information. Because raw distributed acoustic sensing (DAS) data are usually mixed with interference fading, environmental vibrations, and equipment background noise, stable identification of tool operating states can be achieved only after sequential processing steps such as noise suppression, signal decomposition and reconstruction, spatiotemporal feature enhancement, and trajectory extraction. On this basis, image detection, trajectory fitting, and multi-source feature-fusion methods can be further combined to output the continuous position, operating speed, and anomalous state of the tool. The signal acquisition, interpretation, and localization workflow based on distributed fiber-optic sensing is shown in Figure 3.

### 3.1. Noise Suppression in Long-Range Fiber-Optic Sensing

Although distributed fiber-optic sensing offers excellent long-range perception capability, interference fading caused by destructive interference of received light can randomly generate acoustic detection blind zones, thereby constraining the stability of continuous tracking [11,30,31]. To mitigate the impact of such physical dead zones, early studies mainly adopted optical hardware modulation methods such as spatial division multiplexing (SDM) and multi-domain multiplexing (MDM) to reduce the probability of destructive interference and reconstruct effective interference conditions [11,30,32]. On this basis, in order to avoid the high cost of hardware retrofitting, adaptive signal decomposition and statistical filtering methods have developed rapidly. Techniques such as time–distance dual-domain phase differentiation (TDPD) and genetic-algorithm-optimized variational mode decomposition (GA-VMD) can dynamically extract and reconstruct weak vibration features without sacrificing spatial resolution [10,12]. For the construction of long-distance, high-sensitivity vibration-sensing links, fundamental studies on ultra-long-range Phi-OTDR and distributed vibration sensing have provided important system support for subsequent continuous tracking [33,34,35]. In recent years, research has further extended down to the perception-link level, attempting to use deep learning to directly improve the real-time performance of DAS phase demodulation and fine-grained feature recovery, thereby providing higher-fidelity inputs for subsequent localization and identification [36]. Overall, the field has formed a progressive technical route spanning hardware modulation, signal reconstruction, and data-driven enhancement.

### 3.2. Vision-Assisted Interpretation of Distributed Sensing Data

The volume of data generated by distributed fiber-optic sensing systems is enormous and is usually presented in the form of spatiotemporal matrices, or waterfall plots. Traditional threshold-detection methods show obvious limitations, struggle to distinguish the regular vibrations generated by the ILI tool from noise caused by the external environment, and therefore easily lead to misjudgment.

The waterfall plots output by distributed fiber-optic sensing systems provide an intuitive spatiotemporal representation for feature extraction and have driven related research toward the computer-vision paradigm. Compared with traditional one-dimensional energy-threshold methods, which lack robustness under strong background noise, image-based tracking techniques can convert spatiotemporal acoustic-field information into pseudo-color visual images and use target-detection networks such as YOLO to extract global spatial texture features, thereby improving the extraction of nonlinear and variable-speed PIG trajectories under moderate background interference [37]. On this basis, recent work has further focused on the PIG localization task itself. Localization frameworks based on distributed fiber-optic vibration sensing and image segmentation can directly estimate the field position of the PIG through collision-point identification [7]. For scenarios involving superposed multi-source vibrations from vehicles, heavy excavation, and other disturbances, multi-task-learning-based multi-source feature-decoupling methods have established intelligent sensing frameworks for smart DAS (sDAS), achieving separation of the target tool’s acoustic signature through shared low-level feature convolution and dynamically weighted losses [38]. Meanwhile, in related pipeline-sensing studies, lightweight inference technologies for edge deployment have fused horizontal synchrosqueezing transforms with lightweight vision models to realize millisecond-level detection and warning of weak anomalous signals under low-pressure operating conditions [28,39]. However, this result represents transferable evidence and does not by itself establish equivalent end-to-end latency for external PIG localization.

However, the practical performance of vision-assisted DAS interpretation remains limited. Strong and nonstationary background vibrations, such as traffic, construction, and mechanical equipment, can overlap with PIG-induced trajectories, reducing recognition accuracy and increasing the false-positive rate (FPR) [13,38,40].

For long-distance pipelines, even a low-window-level FPR can accumulate into many nuisance alarms due to continuous monitoring [13]. Real-time deployment is also constrained by the high data throughput of DAS systems and the computational demands of image processing and CNN-based inference [39,40].

Mitigation strategies include lightweight models, edge inference, hard-negative training, and multisensor fusion [13,38,39]. However, these approaches may reduce weak-signal sensitivity or localization resolution.

Therefore, YOLO-based methods should be regarded as robustness-enhancing tools rather than complete solutions for long-distance PIG tracking under strong interference.

### 3.3. Hydrophone Arrays and Time-Delay Localization in Cable-Free Deep-Sea Environments

For deep-sea and nearshore areas without parallel communication-optical-cable coverage, noncontact localization based on underwater acoustic sensing networks provides an important complement to fiber-optic continuous tracking in discrete spaces [17]. Complex subsea hydrodynamic reverberation can easily induce severe multipath effects. To address this issue, multi-node time-delay fault-tolerant optimization techniques embed weighted closed-form iterative algorithms, such as WMChan-Taylor, into classical three-dimensional time-difference-of-arrival (TDOA) arrays [41]. Through residual-based statistical adaptation, node signals strongly affected by multipaths and associated with large errors can be suppressed, thereby preserving localization accuracy. The TDOA measurement between the *i*-th and *j*-th hydrophones can be expressed as

$$c\cdot \tau_{ij}={\left\|p-s_{i}\right\|}_{2}-{\left\|p-s_{j}\right\|}_{2},\;\;i\neq j$$

where $p\in R^{3}$ denotes the unknown PIG position; ${s_{i},s}_{j}\in R^{3}$ denote the known positions of the *i*-th and *j*-th hydrophones, respectively; $t_{i}$ and $t_{j}$ are the corresponding signal-arrival times; $\tau_{ij}=t_{i}{-t}_{j}$ is the measured time difference of arrival; and *c* is the effective acoustic propagation speed. The PIG position is estimated by jointly solving the equations obtained from multiple hydrophone pairs.

In practice, TDOA localization accuracy is affected by clock synchronization, multipath propagation, and uncertainty in the effective sound speed. Residual clock offsets or drift between hydrophone nodes introduce systematic errors into $\tau_{ij}$, while reflected and reverberant arrivals may shift or broaden the correlation peak and produce biased time-delay estimates. Generalized cross-correlation with phase transform (GCC-PHAT) weighting and residual-based fault-tolerant estimation can reduce the influence of multipath-contaminated measurements, but they cannot eliminate it completely [41,42]. In addition, errors in *c*, caused by variations in the propagation medium and environmental conditions, are transferred directly into range-difference and position-estimation errors. Accurate implementation therefore requires clock calibration, multipath-robust time-delay estimation, outlier rejection, and site-specific calibration or uncertainty modeling of the effective sound speed. Single-hydrophone approaches that combine multipath time-delay features with particle filtering may reduce the dependence on inter-node clock synchronization, although their performance remains sensitive to multipath interpretation and propagation-model uncertainty [41,42,43].

## 4. Sensor Fusion and AI for Sensor-Data Interpretation and Localization

In deeply buried underground environments or strongly interfered subsea environments, the performance improvement achievable by ILI-tool tracking methods that rely solely on threshold comparison and linear signal processing has become increasingly limited. As a result, recent studies have begun to treat ILI tracking as a sensor-data interpretation problem and have introduced machine-learning methods into pipeline monitoring, leak identification, distributed fiber-optic event classification, and PIG tracking.

From a sensor-centered system perspective, intelligent ILI-tool tracking can be organized into three interconnected layers. The data-acquisition layer consists of magnetic receivers, distributed fiber-optic interrogators, acoustic or pressure sensors, front-end conditioning circuits, analog-to-digital conversion, and timestamp synchronization. The intelligent-modeling layer performs signal denoising, feature extraction, multisensor fusion, and position or state estimation using data-driven and physics-aware models. The engineering-application layer converts the model outputs into tool position, velocity, trajectory, abnormal-operation warnings, and spatial registration of inspection results for pipeline integrity management.

**Figure 4:**
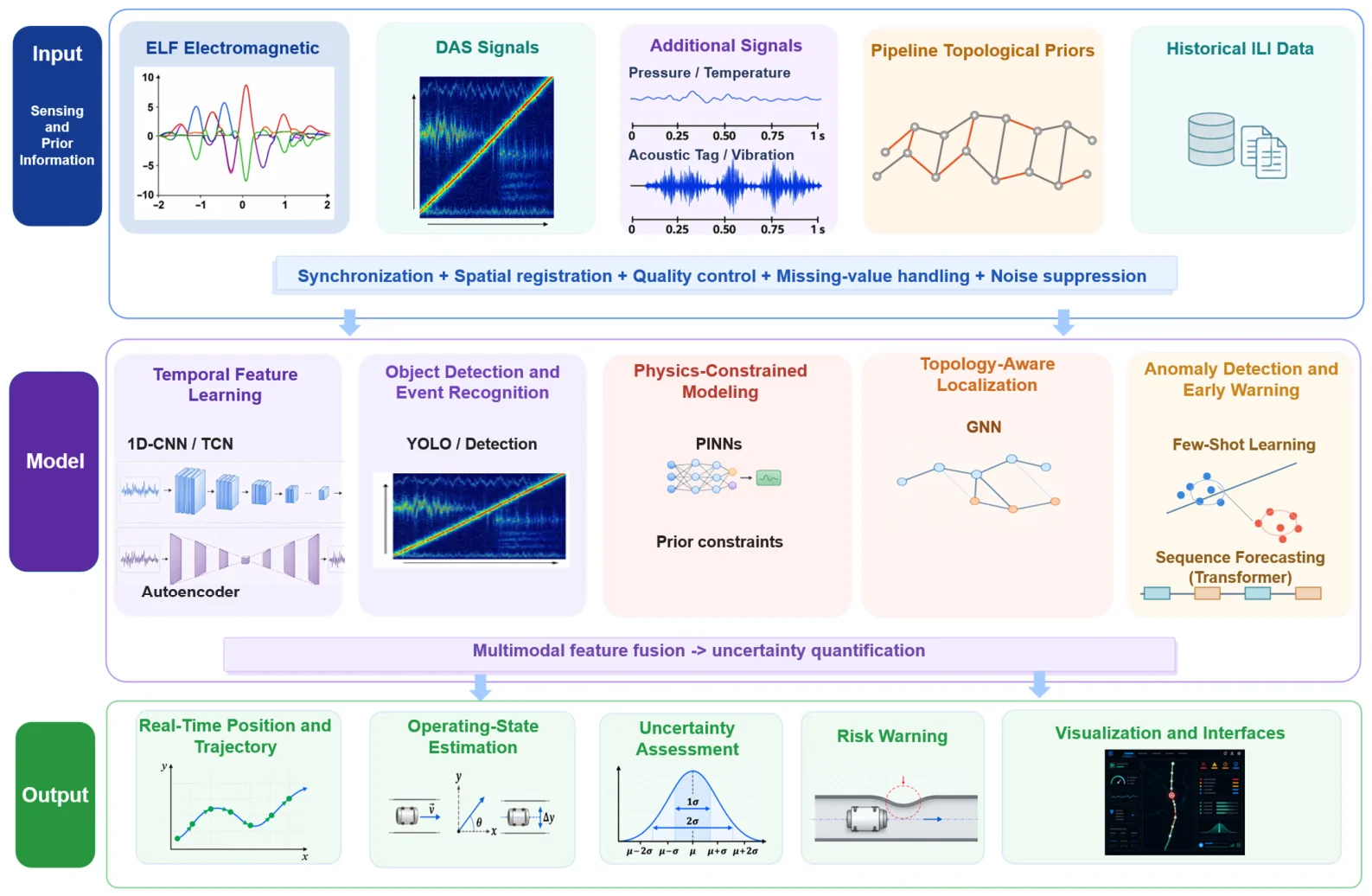
AI-enabled sensor interpretation for tracking and localization.

AI-driven ILI-tool tracking and localization do not rely on a single signal or a single model. Instead, they build a multi-level perception and reasoning framework by fusing ELF electromagnetic signals, distributed fiber-optic vibration signals, pressure and acoustic responses, pipeline-network topology information, and historical ILI data [13,16]. At the model level, temporal convolution and sequence networks can be used to extract transient motion features; target-detection and event-recognition models can be used to detect spatiotemporal trajectories; PINNs can incorporate electromagnetic-field and fluid-transient constraints; and GNNs can describe node associations and path relationships in complex pipeline networks. When further combined with few-shot or unsupervised anomaly detection, these methods can improve the system’s early-warning capability for abnormal operating conditions such as blockage, stagnation, overspeed, and signal loss [44,45]. Representative transferable methods for trajectory warping, physics-informed inversion, and graph-based localization have also begun to emerge in adjacent pipeline-sensing tasks [46,47,48]. In essence, these approaches transform tracking from isolated signal thresholding into multisensor interpretation and localization inference. The relationships among sensing inputs, intelligent models, and localization outputs are shown in Figure 4.

From a practical implementation perspective, sensor fusion can be performed at the state, feature, or decision level. Kalman-filter variants, including ensemble Kalman filtering, can recursively combine position, velocity, and uncertainty estimates when an approximate motion model is available, whereas particle filtering is more suitable for nonlinear or non-Gaussian conditions such as multipath-affected acoustic localization [42,49]. Bayesian methods can further combine heterogeneous measurement likelihoods with prior motion or pipeline-topology constraints. At the feature level, synchronized magnetic, fiber-optic, pressure, and acoustic features can be jointly encoded before localization inference, whereas decision-level fusion combines independently generated detections or position estimates using confidence or reliability weights. In practical systems, fusion performance is affected by timestamp synchronization, sensor calibration and coordinate registration, missing or asynchronous measurements, and disagreement between sensing modalities. Adaptive weighting, outlier rejection, and explicit uncertainty propagation are therefore important when one sensing channel becomes unreliable or temporarily unavailable.

A general multisensor tracking framework can be represented using the following state-transition and observation models:

$$x_{k}=f\left(x_{k-1}\right)+W_{k},$$

$$z_{k}^{\left(m\right)}=h_{m}\left(x_{k}\right)+V_{k}^{\left(m\right)},$$

where $x_{k}$ denotes the state of the ILI tool at time step *k*, which may include its position, velocity, and other operating variables. The measurement vector $z_{k}^{\left(m\right)}$ is obtained from the *m*-th sensing modality, such as magnetic, DAS, acoustic, or pressure sensing. The functions *f(·)* and $h_{m}$*(·)* represent the state-transition model and the modality-specific observation model, respectively, while $W_{k}$ and $V_{k}^{\left(m\right)}$ denote process and measurement noise. This general formulation provides the basis for Kalman filtering, particle filtering, Bayesian estimation, and other multisensor fusion methods.

Existing evidence is stronger for individual sensing modalities than for fully integrated cross-modal PIG-tracking systems. Nevertheless, the complementary coverage and observables of different sensors support several scenario-oriented hybrid architectures. Table 4 summarizes three representative combinations and compares their expected system-level benefits, deployment costs, applicable scenarios, and main limitations.

Table 4 shows that no single hybrid architecture is optimal for all pipeline environments. ELF magnetic–DAS fusion offers continuous coverage and accurate checkpoint correction, but it requires existing fiber infrastructure and additional surface receivers. Hydrophone–fiber fusion provides greater sensing redundancy in subsea environments, although its deployment cost and synchronization requirements are high. Magnetic-pressure/acoustic fusion is more suitable for urban or branched pipeline networks with existing monitoring stations, but its performance is sensitive to operating transients, signal reflections, and network topology. Overall, the selection of a hybrid architecture should balance tracking continuity, localization reliability, deployment cost, infrastructure availability, and environmental conditions. The performance comparison is qualitative and represents expected system-level benefits synthesized from modality-specific studies. Unified benchmarks and directly comparable field data for fully integrated hybrid PIG-tracking systems remain unavailable.

To ensure a consistent interpretation of the reviewed evidence, the methods discussed in this article are classified into three categories according to their relevance to external ILI-tool tracking and localization. Direct evidence refers to methods that have been tested specifically for PIG localization, trajectory extraction, or ILI-related tracking and state estimation. Transferable evidence refers to methods demonstrated in adjacent pipeline-sensing tasks, such as DAS event classification, leak localization, magnetic-flux-leakage evaluation, hydraulic-transient analysis, or pipeline-network state identification. Although these methods may provide relevant sensing principles, physical constraints, or data-processing strategies, their reported performance does not directly establish field performance for external PIG tracking. Prospective approaches refer to emerging methods proposed primarily as future enabling technologies for external PIG tracking and for which direct or closely related validation remains limited. Under this classification, PINNs and GNNs are treated mainly as transferable approaches, whereas digital twins and neuromorphic computing are discussed primarily as prospective directions for external PIG tracking. Accordingly, established magnetic, distributed fiber-optic, and acoustic tracking systems are treated as current engineering solutions, whereas PINNs, GNNs, digital twins, and neuromorphic computing are presented as enabling or prospective technologies whose direct validation in external PIG tracking remains limited.

### 4.1. Data-Driven Decoupling of Transient Time-Series Features and Sequence Prediction

At the stage of temporal-feature decoupling and kinematic-sequence prediction, the key is to separate transient kinematic features associated with ILI tool operation from strong background interference. For one-dimensional sequences such as ELF magnetic signals, distributed fiber-optic vibration signals, and pressure-wave responses, convolutional networks and their variants can automatically extract local temporal features, thereby reducing dependence on manual feature engineering. For sparse anomalies and impulsive arrival fronts commonly found in field data, residual denoising autoencoders and related structures can suppress background noise while preserving abrupt information, thus providing more stable inputs for time-of-arrival estimation and trajectory updating [25,46]. In distributed fiber-optic scenarios, recent studies on event recognition and PIG trajectory extraction have further promoted the real-time engineering deployment of such temporal models [6,7,38,50]. At the same time, studies combining trajectory features with pipeline structural information have shown that kinematic-sequence modeling can also be used jointly with geometric constraints to improve the stability of trajectory recovery [51]. In addition, to address large PIG velocity deviations and significant cumulative mileage errors under complex subsea disturbances, recent studies have begun to introduce multimodal signal decomposition and reconstruction together with AutoRegressive Integrated Moving Average–Residual Long Short-Term Memory (ARIMA-Res-LSTM) hybrid frameworks for state estimation, thereby providing a new modeling idea for robust velocity inversion under complex operating conditions [52].

### 4.2. Dual-Driven Modeling Led by PINNs

Purely data-driven models often suffer from unstable extrapolation and predictions that appear numerically accurate but are physically implausible because they lack physical-law constraints during position regression. This has driven related research to gradually shift toward a dual-driven modeling paradigm led by PINNs. In pipeline scenarios, fluid-transient equations, gas–liquid transport constraints, and magnetic-dipole or magnetic-flux-leakage response models can be embedded into the training process as loss terms, enabling the model to satisfy conservation relationships and field-distribution laws while learning observational data. Direct application of PINNs to external PIG tracking remains limited. Existing evidence is primarily transferable and comes from adjacent applications, including hydraulic-transient analysis in pipeline systems [53], physics-informed magnetic-flux-leakage defect inversion [47], and spectral modeling of transient pipe flow [54]. These studies demonstrate the potential benefits of embedding physical constraints into data-driven models, but they do not establish field performance for external PIG localization. PINNs are therefore discussed here as promising physics-aware frameworks that require direct validation under realistic PIG-tracking conditions.

### 4.3. GNNs for Topology-Aware Sensor Fusion in Complex Pipeline Networks

When dealing with urban underground or subsea pipeline networks featuring numerous branches and complex interconnections, a single linear mileage coordinate is often insufficient to support stable localization, and path ambiguity is especially likely to arise near junctions, valve chambers, and parallel pipe sections. GNNs provide a natural way to represent the association relationships among sensors, pipe sections, and pipeline-network topologies. Their core advantage lies in mapping discrete sensor responses onto graph structures and jointly exploiting local observations and global topological information through node–edge message passing. Current evidence for GNNs is mainly transferable. Existing pipeline applications have focused on leak localization, internal-valve localization, and pipeline-network state identification rather than direct external PIG tracking [48,53,54,55,56,57]. These studies demonstrate that graph-based models can incorporate network topology and node–edge relationships into localization inference. However, their ability to resolve PIG path ambiguity near branches, valve chambers, and parallel pipe sections has not yet been directly validated in operational PIG-tracking scenarios. GNNs should therefore be regarded as topology-aware candidate methods rather than established external PIG-localization solutions.

### 4.4. Few-Shot, Unsupervised, and Cross-Scenario Transfer for Anomaly Early Warning

For low-frequency long-tail events such as tool blockage, abnormal stagnation, overspeed, or sudden signal loss, a supervised-learning framework that depends entirely on large-scale labeled samples is impractical in engineering applications. Compared with the idealized notion of directly identifying anomalies with a zero-shot large model, the currently more feasible route is to use few-shot, semisupervised, or unsupervised anomaly detection together with simulation-to-field transfer learning. On the one hand, samples under normal operating conditions can be used to establish a background distribution, and anomaly alarms can then be triggered when observations deviate from the baseline. On the other hand, simulation data, experimental-loop data, and a small amount of field-labeled data can be combined for cross-scenario transfer to alleviate the shortage of samples under extreme operating conditions. Such methods are more consistent with the way pipeline-monitoring and PIG-operation data are acquired in practice and are also easier to couple with existing DAS, pressure-wave, and magnetic flux leakage (MFL) data links [28,38,44,45].

A major engineering limitation is the extreme scarcity of labeled field data for abnormal PIG conditions, such as blockage, stagnation, and overspeed. Most available datasets come from simulations or controlled pipeline-loop tests. Models trained on these data may generalize poorly to operational pipelines because of differences in geology, burial depth, pipe properties, operating conditions, sensor coupling, and environmental noise. Therefore, high accuracy on simulated data does not necessarily indicate reliable field performance.

Possible solutions include pretraining on simulated data and fine-tuning with limited field samples, semisupervised learning with unlabeled operational data, feature-level domain adaptation, and physics- or digital-twin-assisted data augmentation [44,58,59]. However, direct validation of these methods for abnormal PIG operation remains limited.

### 4.5. Lightweight AI for Edge Deployment

Practical AI-assisted PIG tracking requires lightweight models that can run on resource-constrained edge devices. Low-bit quantization can reduce model size and memory use. Knowledge distillation can transfer useful features from a large model to a smaller one. Lightweight CNNs can also reduce inference time [28,39,60,61].

Event-driven inference offers another option. It activates complex models only when relevant magnetic, acoustic, or DAS features are detected. This can reduce unnecessary computation and power consumption. However, model compression may reduce weak-signal sensitivity and cross-scenario robustness. These methods therefore require direct evaluation under realistic PIG-tracking conditions.

## 5. Challenges and Future Directions for Sensor-Centric Tracking Systems

Despite recent advances, sensor-based ILI-tool tracking and localization still face several overall limitations. These limitations mainly concern sensing performance, intelligent deployment, and system integration. At the sensing level, no single modality can simultaneously provide high sensitivity, wide bandwidth, continuous coverage, low cost, and strong environmental adaptability. This creates fundamental tradeoffs in practical sensor selection. At the intelligence level, limited field data, uncertain model outputs, and constrained computing and energy resources restrict the deployment of advanced algorithms. System-level integration also remains immature, particularly in multi-source data assimilation, uncertainty propagation, digital-twin coupling, and operational-pipeline validation.

Future development should therefore coordinate advances in sensing hardware, edge intelligence, and digital integration. The following subsections discuss the sensitivity–bandwidth tradeoff, edge-computing bottlenecks, and digital-twin-enabled tracking in greater detail. Multimodal sensing, physics-aware fusion, lightweight models, and standardized field benchmarks will be important. Large language models (LLM) may further support high-level interpretation by combining sensor-derived states with pipeline topology, historical inspection records, and maintenance knowledge. However, their role should primarily involve reporting, anomaly interpretation, and decision support, because latency, reliability, data security, and pipeline-specific validation still limit their use in real-time safety-critical localization.

### 5.1. The Sensitivity–Bandwidth Tradeoff

High-sensitivity solid-state magnetic sensors and quantum sensing technologies have expanded sensor-level weak-field measurement capabilities, but their pipeline-level benefits remain constrained by bandwidth and require application-specific validation [15,22,23,24]. When an ILI tool passes a measurement point at high speed in a high-pressure natural-gas pipeline, the spatially coupled magnetic field at the receiver often appears as an extremely narrow transient pulse. Narrowband sensors easily impose a pronounced low-pass filtering effect on such pulses, thereby weakening their advantage in high-frequency spatial resolution [15,23,24]. In the future, heterogeneous integration of high- and low-frequency modalities within multimodal hybrid sensing networks may provide a new path toward resolving this contradiction [28,39].

### 5.2. Edge-Intelligence Bottlenecks

Building on the lightweight AI methods discussed in Section 4.5, their practical deployment remains constrained by computing power, memory, energy consumption, and system architecture.

The introduction of complex spatial optimization and deep networks places higher demands on the computing power and energy management of tracking instruments. In discrete ELF magnetic and acoustic systems, the PIG-side unit, where present, mainly supports signal generation, state recording, or lightweight preprocessing, whereas real-time detection and localization are generally performed at ground receivers or nearby gateways. In distributed fiber-optic systems, data preprocessing and AI inference are typically carried out at the interrogator or a co-located edge server, while hydrophone-array localization is generally completed at a surface buoy, gateway, or shore-based station. Computationally intensive model training and historical-data analysis are more suitable for central servers or cloud platforms. For long-distance pipeline inspection tools that must operate continuously for weeks inside enclosed high-pressure pipe sections, the constraints among computing power, storage, and endurance have become key bottlenecks limiting intelligent deployment. A more realistic future direction for edge intelligence lies in coordinated optimization around lightweight models, knowledge distillation, and event-driven computing architectures, rather than in the simple pursuit of ever larger models [60,61,62].

At the algorithmic level, in addition to conventional low-bit quantization, knowledge distillation has been explored in related pipeline safety-warning tasks to support lightweight edge deployment [60]. Lightweight transfer-learning models have also shown strong performance in pipeline defect diagnosis [61]. However, neither study directly validates external PIG tracking or localization [60,61].

In terms of low-level hardware acceleration, the power-consumption bottleneck of traditional computing architectures has become a major factor constraining further technological development. Given that pipeline ILI tools must operate stably over hundreds of kilometers and that tracking units in most scenarios only need to maintain low-power background listening, this requirement aligns to some extent with the asynchronous event-driven mechanism of spiking neural networks. Neuromorphic computing remains a prospective direction for external PIG tracking. Current support comes mainly from general spiking-neural-network research and structural-health-monitoring applications [62,63], rather than from direct PIG-tracking experiments. Its relevance therefore lies primarily in its potential to support low-power, event-driven processing, and further pipeline-specific validation is required before it can be regarded as an established localization technology.

### 5.3. Digital-Twin-Enabled Tracking and Localization

One important development direction in this field is the deep integration of tracking and localization systems with pipeline digital twins to build virtual counterparts that are linked to physical pipeline networks in real time. Compared with traditional systems that only output single-point coordinates, a digital-twin framework can jointly ingest PIG operating states, external tracking coordinates, distributed fiber-optic vibration signals, pressure data, and historical integrity data to support visual reconstruction, state prediction, and risk-evolution analysis for the tool’s operating process. Existing digital-twin studies have addressed ILI-system workflows, pipeline condition monitoring, risk prediction, and decision-support platforms [64,65,66,67]. These studies provide relevant system-integration concepts, but real-time coupling between a digital twin and external PIG-tracking measurements remains insufficiently validated. Digital twins are therefore treated in this review as prospective cyber–physical integration frameworks rather than field-proven standalone localization methods. Their practical implementation will require reliable multi-source data assimilation, real-time model updating, uncertainty propagation, and validation under operational pipeline conditions [49,67].

## 6. Conclusions

Tracking and localization of oil and gas pipeline ILI tools are increasingly being reshaped as a sensor-system problem rather than a standalone pipeline operation task. This review has synthesized the field from the perspectives of sensing modalities, signal-processing pipelines, and intelligent localization frameworks, covering discrete magnetic tracking, continuous distributed sensing, and AI-enabled interpretation. In this framework, discrete magnetic tracking with centimeter-level localization reported in full-scale controlled tests is represented by [8,9], while continuous distributed sensing and visual localization supported by short field tests are represented by [6,7]. Representative transferable studies on physics-constrained as well as topology-aware intelligent modeling is represented by [47,48].

AI is changing how sensor data are converted into localization decisions, but the relevance and maturity of the available evidence remain uneven. DAS-based ILI-tool trajectory extraction, image-based positioning, and selected ILI-tool state-estimation methods provide direct evidence for specific tracking tasks [6,7,50]. PINNs, GNNs, few-shot learning, and lightweight edge models are supported mainly by transferable evidence from adjacent pipeline-sensing applications [47,48,53,54,55,56,57,60,61]. Digital twins and neuromorphic computing remain primarily prospective directions for external ILI-tool tracking [62,63,64,65,66,67]. These approaches should therefore be interpreted according to their evidence category rather than treated as equally validated localization solutions.

From a system-design perspective, future systems should combine coverage-oriented sensor selection, physics-aware interpretation, uncertainty-aware fusion, and deployment-constrained edge intelligence. Future high-impact work should therefore prioritize shared benchmark datasets for external ILI-tool tracking and localization. Such datasets should contain time-synchronized raw or minimally processed magnetic, distributed fiber-optic, acoustic, pressure, and onboard signals, where available, together with reference ILI-tool position and velocity, sensor timestamps and calibration information, pipeline geometry, burial depth, and representative operating and environmental conditions. Standardized training, validation, and test partitions, common metrics for localization error, detection latency, robustness, and uncertainty, and data from multiple pipeline sections would enable more reliable comparison among algorithms, sensor configurations, and fusion strategies. Where operational data cannot be released, anonymized benchmark subsets or controlled pipeline-loop datasets could provide practical alternatives.

## Figures and Tables

**Table 1:** Streamlined comparison of representative sensing strategies for ILI-tool tracking and localization.

Strategy	Observable	Coverage	Representative Performance/Validation Level	Strength	Vulnerability	Deployment Burden
ELF magnetic	Amplitude; zero crossing; field gradient	Surface checkpoints	Burial depth: 2.5–5 m; PIG speed: 1–15 m/s; centimeter-level localization error reported/ full-scale controlled test [8,9]	High local axial accuracy; burial depth recoverable with multi-receiver layouts	Steel/soil shielding; EM noise	Moderate: surface access and marker deployment
Fiber-optic DAS	Vibration-induced time–distance traces	Full line; km scale	Reported task-specific accuracy: >90%; sensing length: 45 km/ short field test [7]	Continuous trajectory and velocity tracking without marker blind zones	Interference fading; vibration noise; false alarms	High: co-laid fiber and large data throughput
Acoustic/hydrophone	Arrival time; multipath delay	Local/regional; semi-continuous	Pipeline length: 15.9 and 31.3 km/ short field test [17]	Useful where parallel fiber is unavailable, especially subsea	Reverberation; multipath; clock synchronization	High: array synchronization and subsea installation
Hybrid sensor fusion	Joint magnetic, fiber, pressure, and acoustic features	Sensor-stack dependent	No unified benchmark; task-specific metrics/not reported for fully integrated hybrid architectures	Complementary coverage; robust to single-modality failure	Synchronization, calibration, modality disagreement, limited integrated datasets	Very high: calibration, fusion, and annotation overhead

Note: The reported quantitative values and validation settings are study-specific. Validation settings are classified as simulation, laboratory experiment, pipeline-loop test, full-scale controlled test, short field test, or operational-pipeline deployment. Direct comparison is limited by differences in pipeline geometry, burial depth, PIG speed, sensing distance, sensor configuration, performance definitions, and test environments.

**Table 2:** Evolution and engineering characteristics of ELF magnetic sensors for external ILI-tool tracking.

Development Stage	Representative Device	Core Performance Indicator	Main Advantage	Main Limitation	Engineering Status for ILI Tracking
Conventional passive sensing	Induction/search coil	Induced output depends on signal frequency, coil turns, and effective area; weak response in the ELF band	Simple structure; high stability; no excitation power	Large coil size is generally required for weak-signal detection	Mature and widely used in ground receivers
Active magnetic sensing	Fluxgate sensor	Low-frequency noise spectral density and usable bandwidth	Direct measurement of low-frequency and quasi-static magnetic fields	Performance depends on core and excitation design, probe size, bandwidth, and power consumption	Potential option for compact vector sensing
Chip-scale magnetoresistive sensing	TMR sensor	Detectivity and noise spectral density at 1 Hz; 25.3 dB SNR improvement reported with AC modulation and gradiometric measurement [15,22]	Compact size; potentially low power; suitable for vector and gradient arrays	Low-frequency 1/f noise; dependence on bias conditions and readout electronics	Promising, but direct pipeline-level validation remains limited
Quantum magnetic sensing	OPM	Femtotesla-level noise spectral density reported under carefully controlled conditions [23,24]	Extremely high sensor-level magnetic sensitivity	Strict requirements for temperature, optical pumping, field range, shielding, or active compensation	Prospective; pipeline-specific validation remains necessary

**Table 3:** Streamlined comparison of representative ELF weak-signal-processing pipelines (compiled from [8,9,20,21,25,26]).

Pipeline	Core Tool	Complexity	SNR Need	Speed Range	Key Limitation
FFT frequency-domain detection	Steady-state spectral peak extraction	Low	Moderate to high	Low to moderate	Poor resolution in short windows; sensitive to multipath and transient distortion
Fast decision tree (FDT)	Envelope attenuation and peak-width fitting	Very low	Low to moderate	High	Relies on predefined electromagnetic propagation priors; weak generalization
Magnetic field sign integration (MFSI)	Sign-weighted radial/axial field integration	Moderate	Low	Broad speed range	Requires accurate sensor orthogonality and geometry calibration
1D-CNN feature extraction	Latent-feature learning under nonstationary noise	High training/moderate inference	Low	Broad speed range	Model size, data demand, and edge-computing power constraints

Note: SNR, signal-to-noise ratio; 1D-CNN, one-dimensional convolutional neural network.

**Table 4:** Comparison of representative hybrid architectures for external PIG tracking and localization.

Hybrid Architecture	Expected System-Level Benefit	Deployment Cost	Applicable Scenario	Main Limitation
ELF magnetic + DAS	Continuous coverage; checkpoint correction; reduced trajectory drift and false alarms [6,7,8,9].	High	Long-distance onshore pipelines with co-laid fiber	Fiber dependence; surface access; synchronization and registration.
Hydrophone array + fiber sensing	Coverage redundancy; improved availability in fiber-incomplete subsea sections [6,7,17,41,42,43].	Very high	Subsea, offshore, and nearshore pipelines	Multipath; clock synchronization; fiber coupling; underwater installation.
ELF magnetic + pressure/acoustic sensing	Local passage confirmation; regional tracking; improved robustness to weak channels [8,9,17,20,29].	Moderate to high	Urban or branched networks with existing monitoring stations	Reflections; operating transients; topology ambiguity; site-specific calibration.

## Data Availability

No new datasets were generated in this study. All information analyzed in this review is available in the cited publications.
